# Pre hospital care 2012

**DOI:** 10.1186/1757-7241-21-S1-A6

**Published:** 2013-05-28

**Authors:** F Bird, N Pritchard

**Affiliations:** 1 Bartshealth NHS Trust, UK

## 

Recurrent themes ran through the first and second day of London’s trauma conference. The need for identification and effective management of time critical pathology at the point of care was particularly emphasised. Delegates heard of developments in the understanding of the pathophysiology affecting pre-hospital patients, secondary to trauma, cardiac arrest or drowning, and advances in management, from new airway adjuncts to alternative extrication methods and blood product availability.

The second half of the day was dedicated to major incidents and began with a humbling first hand account of the terrorist attacks that affected Norway in 2011. Delegates were reminded of the logistical and medical complexities that result from major incidents and the preparation, planning and training required to deliver an adequate response.

Professor Sir Keith Porter began the day by outlining the background and structure of training for the recently approved UK sub-speciality training programme of pre hospital emergency medicine. Currently the training programme consists of a year (or equivalent) of full time training carried out by senior trainees from the base specialities of anaesthesia or emergency medicine. The programme was developed after a need for consistent high-level advanced medical care to complement paramedic care was identified. It is envisaged that sub-specialists will undertake clinical primary work and inter hospital transfers, take up leadership roles in pre-hospital services and provide medical support to major incidents.

There were four presentations that explored clinical issues in the early phases of care. Dr Lars Wik described the a ‘rapid vehicle extrication technique’ that has been established in Norway. He reminded delegates of the higher mortality rates associated with increased pre-hospital times and the importance of timely extrication in optimising survival. This technique involves the use of chains attached to fixed points at opposite ends of the damaged vehicle, and the application of opposing pulling forces to release the casualty. A paramedic usually remains with the casualty throughout, and the use of cutting instruments may be used as an adjunct. A study that compared the ‘rapid extrication technique’ to the standard technique found significantly shorter times to commencing extrication, faster extrication times and shorter casualty to stretcher times.

Dr Matthew Thomas, a Consultant Anaesthetist from Bristol and Great Western Air Ambulance spoke about supraglottic devices in pre-hospital airway management. He presented evidence that suggests survival rates favour tracheal intubation in some patient groups. He went on to present a case for the role of supraglottic airway devices in pre hospital and trauma settings. He argued that besides the challenges of intubating in a pre-hospital setting and the potential for unrecognised oesophageal intubation, the greatest disadvantage it presents in the cardiac arrest setting is that of interruptions in the provision of CPR. He challenged the theory that intubation is a means of protecting the airway from aspiration by arguing that this, if it is going to occur, will have occurred early, and stressed instead the need for appropriate oxygenation, ventilation and sedation; which he argued can be achieved by a supraglottic device. He discussed the relative merits and disadvantages of different supraglottic airways, suggesting that novel devices (i.e. LMA supreme™ and I-gel™) are likely to be better, and the importance of their use as a rescue technique in the case of a failed intubation. However, he finished by stressing that trained and supported drug assisted tracheal intubation remains a critically important skill.

Dr Bob Winter, President of the Intensive Care Society, spoke about hangings. He gave an informative presentation of the epidemiology, pathophysiology and history behind the classifications of judicial and non-judicial hangings. He explained that the mechanism of injury depends on the type of hanging, of which there are many, but that judicial tends to result in death by spinal transection and non-judicial results in the consequences of compressive injury. Evidence does not support the need for the induction of hypothermia or for C spine immobilisation in casualties of non-judicial incomplete hangings. He did however recommend the use of a lateral C spine X Ray to detect a fractured hyoid – a sign of severe occult tissue injury, a CXR to elicit ARDS - likely caused by negative pressure pulmonary oedema, and potentially the use of CT head and neck (+/- angiogram) to establish the injuries incurred.

Dr Paddy Morgan, Medical Advisor to the RNLI and SLS GB, began his talk by reminding delegates of the large number of deaths attributable to drowning each year; it represents the third leading cause of accidental death worldwide and children are recognised to be particularly at risk (WHO, 2004). He provided a classification for drowning and described the pathophysiology involved, including the additional risks involved on immersion in cold water; that of the cold shock response, and the insidious onset of hypothermia and swim failure. He described the recently published theory of an autonomic conflict seen in victims of face-in immersion that proposes a conflict between the parasympathetic driven diving reflex and sympathetic cold shock response, resulting in potentially fatal cardiac arrhythmias. Dr Morgan described the initial management of a casualty suspected drowning, including the use of ventilatory support with PEEP, and the importance of continued adequate ventilation in the intensive care setting. He reminded delegates of the lessons learnt from the Lyme Bay disaster; the importance of horizontal transfer of casualties from the water, and mentioned the potential future use of artificial surfactant in drowning casualties.

